# A Review: Microbial Diversity and Function of Fermented Meat Products in China

**DOI:** 10.3389/fmicb.2021.645435

**Published:** 2021-06-07

**Authors:** Zhengli Wang, Zhengxi Wang, Lili Ji, Jiamin Zhang, Zhiping Zhao, Rui Zhang, Ting Bai, Bo Hou, Yin Zhang, Dayu Liu, Wei Wang, Lin Chen

**Affiliations:** Key Laboratory for Meat Processing of Sichuan Province, Chengdu University, Chengdu, China

**Keywords:** fermented meat products, microbial diversity, bacteria, fungi, function of microorganisms

## Abstract

Fermented meat products have a long history in China. These products exhibit a characteristic unique flavor, compact meat quality, clear color, long shelf life and wide variety and are easy to transport. During the processing and storage of fermented meat products, microorganisms are present and exhibit diverse characteristics. Microorganisms can accelerate the degradation of proteins and fats to produce flavor compounds, inhibit the growth and reproduction of heterozygous bacteria, and reduce the content of chemical pollutants. This paper reviews the microbial diversity of Chinese ham, sausage, preserved meat, pressed salted duck, preserved fish and air-dried meat and provides analyses of the microbial compositions of various products. Due to the differences in raw materials, technology, auxiliary materials, and fermentation technology, the microbial species found in various fermented meat products in China are different. However, most fermented meat products in China are subjected to pickling and fermentation, so their microbial compositions also have similarities. Microorganisms in fermented meat products mainly include staphylococci, lactobacilli, micrococci, yeasts, and molds. The study of microbial diversity is of great significance for the formation of quality flavor and the safety control of fermented meat products, and it provides some theoretical reference for the study of fermented meat products in China.

## Introduction

Fermented meat products are meat products with longer shelf lives and special flavors, textures and colors that are made of raw meat under specific temperature and humidity conditions by fermentation via microorganisms or enzymes (Liu Y. L. et al., 2020). Fermentation methods include natural fermentation and the artificial addition of a starter to regulate fermentation (Sivamaruthi et al., 2020). The microorganisms involved in the natural fermentation process are primarily derived from raw materials and the environment. Under specific curing and maturing process conditions, typical local microbial communities are formed through competition, thus significantly affecting the product quality and safety (Frédéric et al., 2006). In the 1990s, with the transformation of Chinese people’s demand for meat from quantity to quality, fermented meat products with artificially added microbial starters were gradually introduced into the Chinese market (Yu et al., 2010).

Many traditional meat products, such as Sichuan preserved meat, Hunan preserved meat, Nanjing pressed salted duck, Cantonese sausage, Sichuan sausage, Jinhua ham, Xuanwei ham, and Rugao ham, are all fermented. In recent years, with the improvement of production technology and equipment, research on the flavor, sensory perception, nutritional characteristics and safety of fermented meat products has become a hot topic (Douglas et al., 2020). Microorganisms play an important role during the fermentation of meat products. Flavor formation in fermented meat products is a complex biochemical process that has not been completely understood to date. The microbial diversity of the primary fermented meat products in China is summarized in this paper, and the characteristics of their microbial compositions were analyzed to provide a theoretical basis for further study on fermented meat products.

## The Function of Microorganisms in Fermented Meat Products

Food fermentation is not only an effective method of food preservation but also an economical method of food processing (He et al., 2017). Microorganisms play an important role in fermented meat products, and their functions are as follows:

Promotion of the production of special flavors in meat products (PaPamanoli et al., 2002; Polka et al., 2015). Studies have shown that *Staphylococcus carnosus* (*S. carnosus*) and *Staphylococcus xylosus* (*S. xylosus*) can significantly promote the hydrolysis of fat and protein in preserved meat, improve the color and promote the rapid formation of flavor (Zhou et al., 2018). The esterases, proteases and catalases produced by microorganisms have synergistic effects with endogenous enzymes, causing biochemical changes in the proteins and fats in raw meat and forming small molecules, such as amino acids, esters, peptides and short-chain volatile fatty acids. The digestive function is greatly improved, and the contents of essential amino acids, vitamins and bifidobacterium are increased, enhancing the effects on nutrition and health (Liang et al., 2010). Chen et al. (2017) inoculated Harbin dried sausage with a mixed starter, and the free fatty acid (FFA) content in the inoculated group was significantly higher than that in the uninoculated group. The peroxide value and thiobarbituric acid contents in the inoculated group were significantly reduced compared with those in the uninoculated group, and the contents of aldehydes, ketones and hydrocarbons, which are related to lipid oxidation, were significantly reduced compared with those in the uninoculated group. The mixed starter promoted the hydrolysis of fat, inhibited lipid peroxidation and improved the fermentation flavor of Harbin dried sausage. Wang et al. (2014) added complex microbial starters to traditional preserved meat, and the contents of free amino acids and the types of volatile flavor substances in the preserved meat increased, which improved its nutritional value, flavor and safety.

Inhibition of the growth and reproduction of heterozygous bacteria (Swetwiwathana and Visessanguan, 2015). Microorganisms primarily inhibit the growth and reproduction of harmful bacteria through bacterial competition, acid production and bacteriocin production. Studies have shown that *S. xylosus* can inhibit the growth of *Listeria monocytogenes (L. monocytogenes*) in sausage (PaPamanoli et al., 2002). Wang et al. (2013) inoculated *Latilactobacillus sakei subsp. sakei* (*L. sakei*) as a starter culture into Sichuan sausage, and the results showed that the lactic acid bacteria rapidly controlled the growth of the total bacterial colonies during sausage fermentation and inhibited the growth of foodborne pathogenic bacteria, such as *Escherichia coli* (*E. coli*) and enterobacteriaceae. Lactic acid bacteria can decompose carbohydrates in sausages to produce a large number of lactic acid and a small number of acetic acid as well as propionic acid, formic acid, 3-methyl-butyric acid, butyric acid and other organic acids, thus extending the shelf life of sausages (Li et al., 2015). Lactic acid bacteria can reportedly inhibit or kill spoilage microorganisms and pathogenic microorganisms in food (Zhang et al., 2017). Zhao et al. (2017) isolated and screened a strain of lactic acid bacteria with obvious antibacterial activity against *E. coli* and *Staphylococcus aureus* (*S. aureus*) from five kinds of fermented meat products, which was identified as *Weissella hellenica* (*W. hellenica*). Studies have shown that *L. sakei* C2 can produce broad-spectrum antibacterial bacteriocins and inhibit the growth of harmful microorganisms (Gao et al., 2014).

Reducing the content of biogenic amines (BAs). Sun et al. (2016) found that six types of BAs (cadaverine, putrescine, tryptamine, 2-phenylethylamine, histamine, and tyramine) were inhibited by the presence of *L. plantarum* and *S. xylosus*, and a mixture of these bacteria had the highest inhibitory effect. Niu et al. (2019) selected *L. plantarum* PL-ZL001 from four types of fermented meat products and added it to sausage. PL-ZL001 inhibited the accumulation of six BAs, and its inhibitory effect on the most toxic histamine was significantly greater than that of commercial *S. xylosus*.

Reducing the nitrite content. Wang et al. (2013) inoculated *L. sakei* as a starter culture into Sichuan sausage. The results showed that the nitrite content in the sausage containing added *L. sakei* decreased rapidly from 100 to 9.6 ppm, whereas the nitrite content in the naturally fermented sausage decreased slowly from 100 to 32.1 ppm. You et al. (2015) inoculated lactic acid bacteria into dried salted fish as a starter, and the results showed that the mass fraction of nitrite in the dried salted fish with added lactic acid bacteria was (0.1 ± 0.04) mg⋅kg^–1^, which was significantly lower than that in the group without the addition of lactic acid bacteria at (0.8 ± 0.04) mg/kg^–1^. Lactic acid bacteria had an inhibitory effect on the formation of nitrosamines. Gao et al. (2014) inoculated 5 log CFU/g and 7 log CFU/g *L. sakei* C2 into sausage, and the results showed that the nitrite content decreased with increasing inoculation concentrations.

Microorganisms can improve the quality of fermented meat products to a certain extent. They can not only promote the degradation of protein and fat and accelerate the formation of flavor substances but can also improve the nutritional value of fermented meat products. At the same time, they can inhibit the growth and reproduction of harmful bacteria, reduce the use of salt in fermented meat products, improve the taste, reduce the content of nitrite and BAs, and improve the safety of fermented meat products.

## Microbial Diversity in Fermented Meat Products

### Ham

Ham is a non-ready-to-eat meat product made from fresh (frozen) pig hind legs as the primary raw material with other auxiliary materials add after finishing, curing, washing, desalting, air-drying and fermentation, and other processes. The most famous Chinese-style hams are Jinhua, Rugao, and Xuanwei hams. Alcohols, ketones, aldehydes, esters, alkanes, and acids are considered the primary volatile compounds in hams (Radovčić et al., 2016; Liu D. Y. et al., 2020; Wu et al., 2020). There is a close relationship between the staphylococci and the aldehyde compounds produced as hexanal, nonanal, benzaldehyde, and phenylacetaldehyde, indicating their contributions to the formation of characteristic flavor substances in Jinhua dry-cured ham (Wang Y. B. et al., 2021). Yeasts and molds were primarily present on the surface of Anfu ham, and cocci and yeasts were primarily present inside the ham (Huang et al., 2003). During the dehydration of Jinhua ham, the number of microorganisms inside the ham reached 1.39 × 10^6^ CFU/g, and during the maturation stage, the number of microorganisms inside decreased to 2.0 × 10^3^ CFU/g. Lactic acid bacteria and staphylococci are the dominant bacteria in Jinhua ham. The lactic acid bacteria have primarily been identified as *Pediococcus urinaeequi* (*P. urinaeequi*), *Pediococcus pentosaceus* (*P. pentosaceus*), and *Lactiplantibacillus pentosus* (*L. pentosus*). The staphylococci species primarily include *S. xylosus*, S*taphylococcus equus* (*S. equus*) and *Staphylococcus gallinarum* (*S. gallinarum*) (He et al., 2007). The dominant microorganisms in Xuanwei ham were micrococci, staphylococci and molds, the number of which reached greater than 10^6^ CFU/g on the surface and greater than 10^2^CFU/g inside (Li et al., 2003). Twenty-seven fungi were detected in Xuanwei ham using PCR-DGGE technology. The primary species were *Aspergillus pseudoglaucus* (*A. pseudoglaucus*), *Phialosimplex caninus* (*P. caninus*), *Aspergillus penicillioides* (*A. penicillioides*), *Yamadazyma triangularis* (*Y. triangularis*), *Wallemia sebi* (*W. sebi*), and *Candida glucosophila* (*C. glucosophila*), among which the dominant fungus was *A. pseudoglaucus* (Zou et al., 2020). The diversity and abundance of bacteria in Panxian ham were higher than those of fungi. Ten bacterial phyla were detected at the phylum level, of which Firmicutes and Proteobacteria were dominant. Firmicutes and Proteobacteria were the dominant phyla in the curing process, while Proteobacteria was the dominant phylum in the maturing process. A total of 154 bacterial genera was detected at the genus level, and the dominant genera were *Psychrobacter*, *Acinetobacter*, *Ochrobactrum*, *Staphylococcus*, and *Micrococcus*. Four fungal phyla were detected at the phylum level, and the dominant fungus was Ascomycota. Fifty-one fungal genera were detected at the genus level, and the dominant fungi were *Debaryomyces*, *Aspergillus*, *Yamadazyma*, *Candida*, and *Penicillium* (Mu et al., 2019). After isolation and purification of the dominant microorganisms in Weining ham, a total of 10 strains was obtained, including 4 strains of *Staphylococcus equinosus* (*S. equinosus*), 2 strains of *S. xylosus*, 2 strains of *Lactococcus lactis*, 1 strain of *Candida metapsilosis* (*C. metapsilosis*), and 1 strain of *Candida parapsilosis (C. parapsilosis)* (Zhang et al., 2019). Among the different processing procedures of Nuodeng ham, the bacterial diversity at the salting stage was the highest, and the dominant bacteria at the phylum level were Firmicutes, Proteobacteria, Actinobacteria, and Bacteroidetes. At the genus level, the dominant bacteria included *Psychrobacter, Aeromonas, Shewanella, Pseudomonas*, and *Acinetobacter* (Wang X. R. et al., 2021). The primary species of microorganisms in different hams are presented in Table 1.

**TABLE 1:** Primary microorganisms in different hams.

Type of ham	Primary microorganisms	References
Anfu ham^1^	Skin coat: yeasts, molds Interior: cocci, yeasts	Huang et al., 2003
Jinhua ham^1^	*P. urinaeequi*, *P. pentosaceus*, *L. pentosus*, *S. xylosus*, *S. equus*, *S. gallinarum*	He et al., 2007
Xuanwei ham^2^	*A. pseudoglaucus*, *P. caninus*, *A. penicillioides*, *Y. triangularis*, *W. sebi*, *C. glucosophila*	Zou et al., 2020
Panxian ham^2^	*Psychrobacter*, *Acinetobacter*, *Ochrobactrum*, *Staphylococcus*, *Micrococcus*, *Debaryomyces*, *Aspergillus*, *Yamadazyma*, *Candida*, *Penicilliums*	Mu et al., 2019
Weining ham^2^	*S. equorum*, *S. xylosus*, *P. acidilactici*, *C. metapsilosis*, *C. parapsilosis*	Zhang et al., 2019
Nuodeng ham^2^	*Psychrobacter*, *Aeromonas*, *Shewanella*, *Pseudomonas*, *Acinetobacter*	Wang X. R. et al., 2021

*^1^ Represents the identification of microorganisms by pure culture, and ^2^ represents the identification of microorganisms by molecular biological methods (same below).*

### Sausage

Sausage is a type of non-ready-to-eat meat product made from fresh (frozen) livestock and poultry meat and other auxiliary materials by cutting (or mincing), stirring, curing, filling (or shaping), drying by baking (or drying in the sun, air-drying in the shade), smoking (or not smoking) and other processes. Sausage is primarily produced in Sichuan, Guangdong, Guangxi, Hunan, and Shanghai. The most famous sausages are Cantonese- and Sichuan-style sausages. Sichuan-style sausage is spicy and hot, whereas Cantonese-style sausage is salty and sweet. Among the different styles, Sichuan sausage is distinguished by its high processing and consumption, multiflavored nature and excellent flavor. The flavor components of mature sausage primarily include acetic acid, 3-hydroxy-2-butanone, 2-butanone, esters, volatile phenols, aldehydes and small amounts of alkene, and nitrogen compounds (Hu et al., 2020). Firmicutes, Proteobacteria, Actinobacteria, and Bacteroidetes were detected at the phylum level in Sichuan sausage. Firmicutes (85.65∼93.96%) and Proteobacteria (5.59∼13.95%) were the dominant phyla. *Lactobacillus*, *Weissella*, *Brochothrix*, *Pediococcus*, and *Staphylococcus* were detected at the genus level. Among them, *Weissella* (26.74%) and *Lactobacillus* (63.14%) were the dominant genera. Ninety percent of the fungi in Sichuan sausage were unclassified, and only Bangiales and Ascomycota exceeded 0.1% at the phylum level. *Porphyra* (10.75%) was dominant at the genus level, and *Debaryomyces* and *Saccharomyces* accounted for less than 0.1% (Wang et al., 2019). Nineteen strains of staphylococci and 12 strains of lactic acid bacteria were isolated from Cantonese sausage. According to PCR-DGGE identification, the primary dominant bacteria were *Staphylococcus saprophyticus* (*S. saprophyticus*), *S. xylosus* and *Lactobacillus*, whereas the secondary dominant bacteria were *S. epidermidis*, *Staphylococcus cohnii* (*S. cohnii*), *Lactococcus garvieae* (*L. garvieae*), and *Bacillus* spp. (Xie et al., 2013). At the phylum level, Firmicutes (57.01%), Proteobacteria (30.43%), Cyanobacteria (7.67%), Bacteroidetes (2.63%), and Actinobacteria (2.01%) were present in Hubei Enshi sausage, among which Firmicutes and Proteobacteria were the dominant phyla. At the genus level, *Brochothrix* (38.34%), *Staphylococcus* (9.79%), *Psychrobacter* (7.55%), *Photobacterium* (5.90%), *Pseudomonas* (4.82%), *Lactobacillus* (2.80%), *Leuconostoc* (2.29%), and *Acinetobacter* (2.19%) were identified, among which *Brochothrix* was the dominant genus (Deng et al., 2018). Scholars have studied 16 types of traditional Chinese sausages and found that Firmicutes is the dominant phylum in most sausages. In addition, *Lactobacillus*, *Micrococcus*, *Psychrobacter*, *Tetragenococcus*, and *Pseudomonas* were the dominant genera. The dominant fungal phylum was Ascomycota (Li et al., 2019). Studies have shown that the dominant phyla in five types of northeast sausage are Firmicutes and Proteobacteria, whereas *Lactobacillus*, *Staphylococcus*, *Leuconostoc*, *Lactococcus*, and *Weissella* are the dominant genera. The dominant species are *S. xylosus*, *L. sakei*, *W. hellenica*, *Leuconostoc citreum* (*L. citreum*), *Lactococcus raffinolactis* (*L. raffinolactis*), and *L. plantarum* (Hu et al., 2020). The primary species of microorganisms in different sausages are presented in Table 2.

**TABLE 2:** Primary microorganisms in different sausages.

Type of sausage	Primary microorganisms	References
Sichuan-style sausage^2^	*Lactobacillus*, *Weissella*, *Brochothrix*, *Pediococcus*, *Staphylococcus*, *Porphyra*, *Debaryomyces*, *Saccharomyces*	Wang et al., 2019
Cantonese-style sausage^2^	*S. saprophyticus*, *S. xylosus*, *S. epidermidis*, *S. cohnii*, *L. garvieae*, *Bacillus* spp.	Xie et al., 2013
Enshi sausage^2^	*Brochothrix*, *Staphylococcus*, *Psychrobacter*, *Photobacterium*, *Pseudomonas*, *Lactobacillus*, *Leuconostoc*, *Acinetobacter*	Deng et al., 2018
16 types of traditional sausage^2^	*Micrococcus*, *Psychrobacter*, *Tetragenococcus*, *Pseudomonas*, *Debaryomyces*, *Wallemia*, *Aspergillus*	Li et al., 2019
Sausages from Northeast China^2^	*S. xylosus*, *L. sakei*, *W. hellenica*, *L. citreum*, *L. raffinolactis*, *L. plantarum*	Hu et al., 2020

*^2^ Represents the identification of microorganisms by molecular biologica methods.*

### Preserved Meat

Preserved meat is a type of non-ready-to-eat meat product made from fresh (frozen) livestock meat as the primary raw material, along with other auxiliary materials by curing, drying by baking (or drying in the sun, air-drying in the shade), smoking (or not smoking), and other processes. Preserved meat is primarily popular in Sichuan, Hunan, and Guangdong. Hexanal, 3-methyl butyraldehyde, 3-methyl valeraldehyde, (E)-2-octenal, octanal, linalool, nonanal, hexanoic acid, ethyl hexanoate, anisole, and acetone were the primary flavor contributors in preserved meat (Guo et al., 2020; Mao et al., 2021). The bacteria at the phylum level in Sichuan traditional preserved meat include Firmicutes, Proteobacteria, Actinobacteria, Cyanobacteria, and Bacteroidetes, and the dominant phylum is Firmicutes. At the genus level, the bacteria included *Staphylococcus*, *Micrococcus*, *Acinetobacter*, *Psychrobacter*, *Pseudomonas*, *Brochothrix*, *Cupriavidus*, *Citrobacter*, and *Enterobacter*, and the dominant genus was *Staphylococcus*. The fungi at the phylum level in Sichuan traditional preserved meat include Ascomycota, Basidiomycota, Glomeromycota, and Rozellomycota, and the dominant phylum was Ascomycota. At the genus level, *Aspergillus*, *Debaryomyces*, *Candida*, *Wallemia*, *Penicillium*, *Malassezia*, and *Didymella* were dominant, and the dominant fungus was *Aspergillus* (Wen et al., 2020). The dominant microorganisms during the shelf life of Sichuan preserved meat were *Staphylococcus* and *Micrococcus* followed by *Lactobacillus* (Quan et al., 2017). At the phylum level, the microorganisms in Hubei Enshi preserved meat primarily included Firmicutes (54.05%) and Proteobacteria (44.28%), while at the genus level, the microorganisms were primarily *Staphylococcus* (40.18%), *Psychrobacter* (24.02%), *Pseudoalteromonas* (9.37%), *Brochothrix* (8.53%), *Cobetia* (4.71%), and *Acinetobacter* (2.31%) (Deng et al., 2018). The number of yeasts and molds in Xiangxi preserved meat in Hunan Province reached 2.6 × 10^7^ CFU/g. The number of staphylococci and micrococci reached 3.7 × 10^6^ CFU/g. The number of lactic acid bacteria reached 2.4 × 10^6^ CFU/g, and the number of heat-resistant microorganisms reached 3.5 × 10^3^CFU/g (Dong et al., 2018). A study on the microbial diversity of preserved meats in Hunan Province found that the microorganisms included approximately 20 phyla, among which Firmicutes, Proteobacteria, Cyanobacteria, and Actinobacteria were the dominant phyla. In addition, there were more than 10 microbial genera, among which *Staphylococcus*, *Sphingomonas*, *Pseudomonas*, *Enterobacter*, and *Leuconostoc* were the dominant genera (Yi et al., 2017). The lactic acid bacteria in Longxi preserved meat included *L. plantarum*, *Latilactobacillus curvatus* (*L. curvatus*), *L. sakei*, *Companilactobacillus alimentarius* (*C. alimentarius*), *P. pentosaceus*, and *Leuconostoc mesenteroides* (*L. mesenteroides*), of which *L. plantarum* has strong acid production capacity and is quite tolerant to sodium chloride and sodium nitrite (Deng et al., 2019). The total microbial number in Jilin Changchun preserved meat was 2.863 × 10^7^ CFU/g. At the genus level, before air drying, the dominant microorganisms included *Bacillus*, *Acinetobacter*, and *Halospirulina*. After air drying for 1 week, the dominant microorganisms were *Pseudomonas*, *Acinetobacter*, *Psychrobacter*, and *Halospirulina*. After air drying for 2 weeks, the dominant microorganisms were *Erwinia*, *Halospirulina*, *Enterobacter*, and *Acinetobacter*. After air drying for 3 weeks, the dominant microorganisms included *Bacillus*, *Halospirulina*, *Enterobacter*, and *Erwinia*. After air drying for 4 weeks, the dominant microorganisms were *Halospirulina*, *Acinetobacter*, *Enterobacter*, and *Microbacteriaceae* (Li et al., 2020). The primary microorganisms species in different preserved meats are presented in Table 3.

**TABLE 3:** Primary microorganisms in different preserved meats.

Type of preserved meat	Primary microorganisms	References
Sichuan preserved meat^2^	*Staphylococcus*, *Micrococcus*, *Acinetobacter*, *Psychrobacter*, *Pseudomonas*, *Brochothrix*, *Cupriavidus*, *Citrobacter*, *Enterobacter*, *Aspergillus*, *Debaryomyces*, *Candida*, *Wallemia*, *Penicillium*, *Malassezia*, *Didymella*	Wen et al., 2020; Quan et al., 2017
Enshi preserved meat^2^	*Staphylococcus*, *Psychrobacter*, *Pseudoalteromonas*, *Brochothrix*, *Cobetia*, *Acinetobacter*	Deng et al., 2018
Hunan preserved meat^1^	*Staphylococcus*, *Sphingomonas*, *Pseudomonas*, *Enterobacter*, *Leuconostoc*	Yi et al., 2017
Longxi preserved meat^1^	*L. plantarum*, *L. curvatus*, *L. sakei*, *C. alimentarius*, *P. pentosaceus*, *L. mesenteroides*	Deng et al., 2019

*^1^ Represents the identification of microorganisms by pure culture, and ^2^ represents the identification of microorganisms by molecular biological methods.*

### Pressed Salted Duck

Pressed salted duck is a non-ready-to-eat meat product made from duck as the raw material processed by slaughter, hair removal, evisceration, cleaning and curing, shaping, and air drying. Pressed salted duck is a specialty of the southwest region, and famous products include Chongqing Baishiyi pressed salted duck, Nanan pressed salted duck, and Jianchang pressed salted duck. Benzaldehyde and (E,E)-2,4-nonadienal are the key flavor compounds of pressed salted duck. Hexanal, nonanal, naphthalene, (Z)-2-heptenal, (E)-2-octenal, (E)-2-kunienal, 1-octene-3-ol, 2-n-pentylfuran and linalool were the primary compounds affecting the flavor differences in pressed salted ducks from different regions (Tong et al., 2018). The total number of microbial colonies in Sichuan air-dried ducks was 4.692 × 10^3^ CFU/g, and *Neisseria*, *Micrococcus*, and *Staphylococcus* were the primary dominant genera (Liu et al., 2007). The beneficial microorganisms in Jianchang pressed salted duck primarily include staphylococci, lactic acid bacteria, micrococci, yeasts, and molds. The staphylococci include *S. carnosus*, *Staphylococcus simulans* (*S. simulans*), and *S. xylosus*. The lactic acid bacteria include *Lactobacillus* [*L. plantarum*, *L. sakei*, *Lactobacillus acidophilus* (*L*. *acidophilus*), *L. lactis*, *Lacticaseibacillus casei* (*L. casei*), *L. curvatus*, *Limosilactobacillus fermentum* (*L. fermentum*), *Loigolactobacillus bifermentans* (*L. bifermentans*), *Levilactobacillus brevis* (*L. brevis*), *Lentilactobacillus buchneri subsp. buchneri* (*L. buchneri*), *Lactobacillus helveticus* (*L. helveticus*), and *Lactobacillus delbrueckii subsp. bulgaricus* (*L. delbrueckii*)], *Streptococcus* [*Streptococcus lactis* (*S. lactis*), *Streptococcus acidilactis* (*S. acidilactis*), *Streptococcus diacetilactis* (*S. diacetilactis*)] and *Pediococcus* [*Pediococcus acidilactici* (*P. acidilactici*), *P. pentosaceus*, and *Pediococcus cerevisiae* (*P. cerevisiae*)]. The micrococci include *Micrococcus auterisiae* (*M. auterisiae*), *Micrococcus candidus* (*M. candidus*), *Micrococcus varians* (*M. varians*), *Micrococcus roseus* (*M. roseus*), *Micrococcus epidermidis* (*M. epidermidis*), and *M. luteus* (Lin, 2017). The predominant spoilage bacteria in water-cooked salted duck at the end of the shelf life were *Brochothrix thermosphacta* (*B. thermosphacta*) and lactic acid bacteria, and minor components were enterobacteriaceae, micrococci, yeasts and molds (Li et al., 2010). The primary species of microorganisms in different pressed salted ducks are presented in Table 4.

**TABLE 4:** Primary microorganisms in different pressed salted ducks.

Type of pressed salted duck	Primary microorganisms	References
Sichuan air-dried duck^1^	*Neisseria*, *Micrococcus*, *Staphylococcus*	Liu et al., 2007
JianChang pressed salted duck^2^	*S. carnosus*, *S. simulans*, *S. xylosus*, *L. plantarum*, *L. sakei*, *L. acidophilus*, *L. lactis*, *L. casei*, *L. curvatus*, *L. fermentum*, *L. bifermentans*, *L. brevis*, *L. buchneri*, *L. helveticus*, *L. delbrueckii.*, *S. lactis*, *S. acidilactis*, *S. diacetilactis*, *P. acidilactici*, *P. pentosaceus*, *P. cerevisiae*, *M. auterisiae*, *M. candidus*, *M. varians*, *M. roseus*, *M. epidermidis*, *M. luteus*, yeasts, molds	Lin, 2017
Water-cooked salted duck^1^	*B. thermosphacta*, lactic acid bacteria, enterobacteriaceae, micrococci, yeasts, molds	Li et al., 2010

*^1^ Represents the identification of microorganisms by pure culture, and ^2^ represents the identification of microorganisms by molecular biological methods.*

### Preserved Fish

Preserved fish are made from fresh fish that are processed by slaughter and scale removal, evisceration, curing, and drying by baking (or drying in the sun, air-drying in the shade). Preserved fish are characterized by their unique flavor and storage stability (Hu and Wang, 2018). The volatile flavor compounds in traditional preserved fish are primarily aldehydes, alcohols and heterocyclic compounds. Among them, hexanal, caprylic aldehyde, nonyl aldehyde, 2-non-ene aldehyde, decanal, 3-methyl butyraldehyde, (z)-4-heptylaldehyde, benzaldehyde, 1-octene-3-ol, 1-pentene-3-ol, 3-methyl butanol, heptanol, trimethylamine, and 3-methyl-1H-indole are the primary active substances (Gu et al., 2019; Xu et al., 2020; Zhang et al., 2020). Microbes at the phylum level in Enshi-preserved fish from Hubei included Proteobacteria (61.29%), Firmicutes (30.21%), Bacteroidetes (5.34%), and Actinobacteria (1.74%). At the genus level, the primary genera included *Psychrobacter* (35.70%), *Brochothrix* (19.74%), *Pseudomonas* (7.13%), *Staphylococcus* (7.12%), *Acinetobacter* (4.19%), *Vibrio* (3.90%), *Pseudoalteromonas* (3.09%), and *Chryseobacterium* (1.98%) (Wang et al., 2018). Zeng et al. (2009) found that the dominant microorganisms during the processing of preserved fish were lactic acid bacteria, micrococci, staphylococci, and yeasts. The dominant microorganisms at the phylum level during the processing of dried preserved fish included Bacteroidetes, Firmicutes and Proteobacteria. At the family level, the dominant microorganisms largely included Xanthomonadaceae, Comamonadaceae, Campylobacteraceae, Clostridiaceae, Lacto bacteriaceae, Vibrionaceae, Streptococcaceae, Aeromonadaceae, Moraxellaceae, Planococcaceae, Shewanellaceae, Entero coccaceae, Pseudomonadaceae, Staphylococcaceae, Bacillaceae, and Enterobacteriaceae (Wu et al., 2017). The primary species of microorganisms in different preserved fish are presented in Table 5.

**TABLE 5:** Primary microorganisms in different preserved fish.

Type of preserved fish	Primary microorganisms	References
Enshi preserved fish^2^	*Psychrobacter*, *Brochothrix*, *Pseudomonas*, *Staphylococcus*, *Acinetobacter*, *Vibrio*, *Pseudoalteromonas*, *Chryseobacterium*	Wang et al., 2018
Preserved fish^2^	Lactic acid bacteria, micrococci, staphylococci, yeasts	Zeng et al., 2009
Salted dried fish^2^	Xanthomonadaceae, Comamonadaceae, Campylobacteraceae, Clostridiaceae, Lactobacteriaceae, Vibrionaceae, Streptococcaceae, Aeromonadaceae, Moraxellaceae, Planococcaceae, Shewanellaceae, Enterococcaceae, Pseudomonadaceae, Staphylococcaceae, Bacillaceae, Enterobacteriaceae	Wu et al., 2017

*^2^ Represents the identification of microorganisms by molecular biologica methods.*

### Air-Dried Meat

Air-dried meat is stored at temperatures below zero. The meat is cut into small strips and treated with spices, pickled and hung in the shade, and it is naturally air-dried for approximately 3 months to form a directly edible product. After drying, the meat is crispy, and this product is commonly found in Xizang and northwestern Inner Mongolia. The volatile flavor compounds in air-dried meat include acids, aldehydes, ketones, alcohols, alkenes, sulfur-containing compounds and heterocyclic compounds, among which heptanal, 1-octene-3-ol, cyclopentanol, 3-hydroxy-2-heptanone, and 6-methyl-5-heptene-2-ketone are the primary contributors (Ma et al., 2021). The dominant microorganisms during the air-drying process of Xinjiang air-dried beef were lactic acid bacteria, staphylococci and micrococci. The total number of microorganisms was 8.0 × 10^8^ CFU/g, the number of lactic acid bacteria was 1.48 × 10^7^ CFU/g, the number of staphylococci and micrococci was approximately 4.40 × 10^8^ CFU/g, the number of enterobacteria was approximately 10^2^ CFU/g, the number of enterococci was less than 10^5^ CFU/g, and the number of yeasts and molds was approximately 1.50 × 10^4^ CFU/g (Wang et al., 2016). Lei et al. (2017) found 36% enterobacteria, 33% yeasts, 16% pseudomonades, 10% cocci, and 5% lactic acid bacteria in Xinjiang air-dried beef. In naturally fermented air-dried meat, 21 microbial phyla and 241 microbial genera were detected, and the dominant phyla included Firmicutes (39%), Proteobacteria (40%), and Bacteroidetes (14%) (Tian et al., 2019). The primary species of microorganisms in different air-dried meat samples are presented in Table 6.

**TABLE 6:** Primary microorganisms in different air-dried meat.

Type of air-dried meat	Primary microorganisms	References
Xinjiang air-dried beef^1^	Lactic acid bacteria, staphylococci, micrococci, enterobacteria, pseudomonades, enterococci, molds, yeasts, cocci	Wang et al., 2016; Lei et al., 2017
Naturally fermented air-dried meat^2^	Firmicutes, Proteobacteria, Bacteroidetes	Tian et al., 2019

*^1^ Represents the identification of microorganisms by pure culture, and ^2^ represents the identification of microorganisms by molecular biological methods.*

## Conclusion and Prospect

Due to differences in raw materials, technology, auxiliary materials and fermentation time, the microbial species in fermented meat products in China are different. However, most fermented meat products in China are subjected to salting and fermentation, so the microbial compositions share some similarities. Microorganisms in fermented meat products mainly include staphylococci, lactobacilli, micrococci, yeasts, and molds.

At present, research on fermented meat products largely focuses on the microbial diversity of fermented meat products, the mechanisms by which specific microbes influence the quality of fermented meat products, the characteristic flavor of fermented meat products and their production pathway. A large number of studies have shown that microorganisms can promote the formation flavor of fermented meat products and inhibit the generation of BAs, nitrosamines and other harmful chemicals in fermented meat products, but the specific mechanisms have not been fully reported. In the future, research on these mechanisms will provide effective theoretical support for improving the production process and the modernization and standardization of production of fermented meat products.

## Author Contributions

ZLW and ZXW conducted the literature collection and literature research. ZLW drafted the original manuscript. ZLW, ZXW, and LC modified the manuscript. All authors critically reviewed, contributed to, and approved the final manuscript.

## Conflict of Interest

The authors declare that the research was conducted in the absence of any commercial or financial relationships that could be construed as a potential conflict of interest.

## References

[B1] Chen Q. Kong B. H. Han Q. Xia X. F. Xu L. (2017). The role of bacterial fermentation in lipolysis and lipid oxidation in Harbin dry sausages and its flavour development. *LWT Food Sci. Technol.* 77 389–396. 10.1016/j.lwt.2016.11.075

[B2] Deng F. Wang Y. R. Shang X. J. She M. N. Ge D. Y. Zhao H. J. (2018). Bacterial diversity of sausages in Enshi of Hubei province. *Meat Res.* 32 18–22. 10.7506/rlyj1001-8123-201809004

[B3] Deng Z. R. Yun J. M. Guo J. Niu Y. X. Li Y. H. (2019). Study on isolation and fermentation performance of dominant lactic acid bacteria during the processing of Longxi Bacon. *Curr. Biotechnol.* 9 200–209. 10.19586/j.2095-2341.2018.0098

[B4] Dong Y. Wang Y. R. Wang Y. Liao H. Zhao H. J. Guo Z. (2018). Evaluation of bacterial diversity in Chinese bacon from enshi by denatured gradient gel electrophoresis and MiSeq high-throughput sequencing. *Meat Res.* 32 37–42. 10.7506/rlyj1001-8123-201810007

[B5] Douglas P. Erick S. Manuel L. J. Mirian P. Ruben D. Alves D. S. B. (2020). Low-sodium dry-cured rabbit leg: a novel meat product with healthier properties. *Meat Sci.* 173:108372. 10.1016/j.meatsci.2020.108372 33229105

[B6] Frédéric L. Jurgen V. Luc D. V. (2006). Functional meat starter cultures for improved sausage fermentation. *Intern. J. Food Microbiol.* 106 270–285. 10.1016/j.ijfoodmicro.2005.06.027 16213053

[B7] Gao Y. R. Li D. P. Liu X. Y. (2014). Bacteriocin-producing *Lactobacillus sakei* C2 as starter culture in fermented sausages. *Food Control* 35 1–6. 10.1016/j.foodcont.2013.06.055

[B8] Gu S. Q. Tang J. J. Zhou X. X. Zheng H. M. Zhou H. X. Ding Y. T. (2019). Quality change and aroma formation in cured fish during traditional sun drying processing. *Food Sci.* 40 36–44. 10.7506/spkx1002-6630-20180716-201

[B9] Guo J. Wang Q. Chen C. G. Yu H. Xu B. (2020). Effects of different smoking methods on sensory properties, free amino acids and volatile compounds in bacon. *J. Sci. Food Agric.* 101 2984–2993. 10.1002/jsfa.10931 33159340

[B10] He G. Q. Liu T. J. Sadiq F. A. Gu J. S. Zhang G. H. (2017). Insights into the microbial diversity and community dynamics of Chinese traditional fermented foods from using high-throughput sequencing approaches. *J. Zhejiang Univ. Sci. B* 18 289–302. 10.1631/jzus.B1600148 28378567 PMC5394094

[B11] He Z. F. Li H. Y. Yu X. Zhou R. H. Zhang M. (2007). Study on the changes in microbial flora of jinhua ham in modern fermentation technology. *J. Southwest Univ.* 29 142–150. 10.3969/j.issn.1673-9868.2007.03.030

[B12] Hu D. Wang M. (2018). Research on processing technology of Chinese traditional preserved fish. *Technol. Econ. Guide* 26:61.

[B13] Hu Y. Y. Zhang L. Liu Q. Wang Y. Chen Q. Kong B. H. (2020). The potential correlation between bacterial diversity and the characteristic volatile flavour of traditional dry sausages from Northeast China. *Food Microbiol.* 91:103505. 10.1016/j.fm.2020.103505 32539975

[B14] Huang Z. W. Xu M. S. Tang K. J. Jiang Y. Wu S. F. (2003). Separation of fermentation microorganism in Aufu Ham and its fermentation technology. *Acta Agric. Univ. Jiangxien.* 25 635–638. 10.3969/j.issn.1000-2286.2003.04.037

[B15] Lei Y. H. Guo J. N. Sun B. Z. Xie P. Zhang Y. Li H. B. (2017). Characteristics of microbial changes in the processing of dried beef in Xinjiang. *J. Food Saf. Q.* 8 2914–2921. 10.3969/j.issn.2095-0381.2017.08.013

[B16] Li J. X. Wang W. Wang X. H. Bai T. (2015). The source and formation of flavor substances in fermented sausage. *Food Sci. Technol.* 40 160–165. 10.13684/j.cnki.spkj.2015.05.032

[B17] Li L. Zou D. Ruan L. Y. Wen Z. Y. Chen S. W. Xu L. (2019). Evaluation of the biogenic amines and microbial contribution in traditional Chinese sausages. *Front. Microbiol.* 10:872. 10.3389/fmicb.2019.00872 31130922 PMC6510162

[B18] Li P. L. Shen Q. W. Lv Y. N. Jiang Z. J. Ma C. W. (2003). Analysis of main of microorganisms in Xuanwei Ham. *Chin. J. Microecol.* 15 12–13. 10.3969/j.issn.1005-376X.2003.05.007

[B19] Li X. R. Liu L. Y. Yang Y. Hou P. P. Zhao Y. T. Zhang T. H. (2020). Physicochemical, microbial and flavor profiles of traditional Chinese cured meat. *Meat Res.* 34 22–26. 10.7506/rlyj1001-8123-20190821-187

[B20] Li Y. L. Yao D. R. Wang D. Y. Xu W. M. Zhu Y. Z. Jin B. Q. (2010). Sensory, physicochemical and microbiological changes in water-cooked salted duck during storage at 4°C. *Asian Austr. J. Anim. Sci.* 23 960–964. 10.5713/ajas.2010.90550

[B21] Liang J. Zhao J. R. Ji Y. G. Sun S. L. Zhan J. F. (2010). Microbiological safety analysis of several fermented meat products. *Heilongjiang Anim. Sci. Vet. Med.* 52, 34–35. 10.13881/j.cnki.hljxmsy.2010.22.002

[B22] Lin Q. (2017). Jianchang Duck in the fermentation process of microorganism. *Modern Food* 33 66–69. 10.16736/j.cnki.cn41-1434/ts.2017.06.027

[B23] Liu D. Y. Bai L. Feng X. Chen Y. P. Zhang D. N. Yao W. S. (2020). Characterization of Jinhua ham aroma profiles in specific to aging time by gas chromatography-ion mobility spectrometry (GC-IMS). *Meat Sci.* 168:108178. 10.1016/J.MEATSCI.2020.108178 32417671

[B24] Liu X. Zhang W. X. Yue Y. Y. Wang W. Yu H. (2007). A preliminary study on the microflora of Wind-blown duck from Sichuan. *Sci. Technol. Food Indust.* 28, 82–87. 10.3969/j.issn.1002-0306.2007.07.022

[B25] Liu Y. L. Yu Q. L. Wan Z. Li H. Y. Liu J. Wang J. (2020). Research progress of antioxidant activity of starter culture on the quality of fermented meat products. *Food Sci.* 82 1–17. 10.7506/spkx1002-6630-20200704-052

[B26] Ma G. L. Tang S. H. Li S. N. Liu H. L. Ren R. (2021). Changes of physicochemical properties and volatile flavor substances in tibetan air-dried yak meat jerky during the simulated processing. *Sci. Technol. Food Industr.* 42 19–25. 10.13386/j.issn1002-0306.20200301018

[B27] Mao Y. Q. Li Y. H. Yun J. M. He K. Wang R. Wu S. J. (2021). Volatile flavor compounds in traditional Longxi bacon production. *Food Fermentat. Industr.* 47 144–152. 10.13995/j.cnki.11-1802/ts.025248

[B28] Mu Y. Su W. Mu Y. C. Jiang L. (2019). Combined application of high-throughput sequencing and metabolomics reveals metabolically active microorganisms during Panxian Ham processing. *Front. Microbiol.* 10:3012. 10.3389/fmicb.2019.03012 31998279 PMC6966718

[B29] Niu T. J. Chen L. S. Kong H. G. Guo Y. J. Ma Y. (2019). Screening, identification and application of biogenic amine degrading strains derived from traditional fermented meat products. *China Brew.* 38 43–48. 10.11882/j.issn.0254-5071.2019.09.009

[B30] PaPamanoli E. Kotzekidou P. Tzanetakis N. Litopoulou-Tzanetaki E. (2002). Characterization of Mieroeoccaeeae isolated from dry fermented sausage. *Food Microbiol.* 19 441–449. 10.1006/fmic.2002.0503 22063449

[B31] Polka J. Rebecchi A. Pisacane V. Morelli L. Puglisi E. (2015). Bacterial diversity in typical Italian salami at different ripening stages as revealed by high-throughput sequencing of 16S rRNA Amplicons. *Food Microbiol.* 46 342–356. 10.1016/j.fm.2014.08.023 25475305

[B32] Quan T. Deng D. C. Li H. J. He Z. F. Zhang Y. H. Zhang J. X. (2017). Study on the main microorganisms of traditional Sichuan bacon during its shelf life. *J. Southwest Univ.* 39 14–21. 10.13718/j.cnki.xdzk.2017.02.003

[B33] Radovčić N. M. Vidaček S. Janči T. Medić H. (2016). Characterization of volatile compounds, physico-chemical and sensory characteristics of smoked dry-cured ham. *J. Food Sci. Technol.* 53 4093–4105. 10.1007/s13197-016-2418-2 28035165 PMC5156653

[B34] Sivamaruthi B. S. Kesika P. Chaiyasut C. (2020). A narrative review on biogenic amines in fermented fish and meat products. *J. Food Sci. Technol.* 58 1623–1639. 10.1007/s13197-020-04686-x 33897002 PMC8021659

[B35] Sun Q. X. Chen Q. Li F. F. Zheng D. M. Kong B. H. (2016). Biogenic amine inhibition and quality protection of Harbin dry sausages by inoculation with *Staphylococcus xylosus* and *Lactobacillus plantarum*. *Food Control* 68 358–366. 10.1016/j.foodcont.2016.04.021

[B36] Swetwiwathana A. Visessanguan W. (2015). Potential of bacteriocin-producing lactic acid bacteria for safety improvements of traditional Thai fermented meat and human health. *Meat Sci.* 109 101–105. 10.1016/j.meatsci.2015.05.030 26100576

[B37] Tian J. J. Zhang K. P. Yang M. Y. Jing Z. B. Li Q. W. Zhao L. H. (2019). Comparative bacterial diversity analysis and microbial safety assessment of air-dried meat products by Illumina MiSeq Sequencing technology. *Food Sci.* 40 33–40. 10.7506/spkx1002-6630-20180504-043

[B38] Tong H. G. Wang W. Zhang H. F. Li P. J. Chen C. G. Chen J. (2018). Comparative analysis flavor components of dry-cured ducks from different regions by HPLC, GC-MS combined with multivariate statistical analysis. *Modern Food Sci. Technol.* 34 228–238. 10.13982/j.mfst.1673-9078.2018.12.034

[B39] Wang J. G. Li Y. H. Guo A. M. Han D. Y. Liu C. J. (2016). Physico-chemical and microbial properties of Xinjiang dry-cured beef during the ripening process. *Food Fermentat. Industr.* 42 129–133. 10.13995/j.cnki.11-1802/ts.201610022

[B40] Wang W. Liu Y. Wang X. H. Zhang J. M. (2014). Effects of microbial fermentation agents on the storable nature and flavor characteristics of Sichuan bacon. *Food Sci. Technol.* 39 159–164. 10.13684/j.cnki.spkj.2014.09.035

[B41] Wang X. H. Ren H. Y. Liu D. Y. Zhu W. Y. Wang W. (2013). Effects of inoculating *Lactobacillus sakei* starter cultures on the microbiological quality and nitrite depletion of Chinese fermented sausages. *Food Control* 32 591–596. 10.1016/j.foodcont.2013.01.050

[B42] Wang X. H. Wang S. H. Zhao H. (2019). Unraveling microbial community diversity and succession of Chinese Sichuan sausages during spontaneous fermentation by high-throughput sequencing. *J. Food Sci. Technol.* 56 3254–3263. 10.1007/s13197-019-03781-y 31274892 PMC6582033

[B43] Wang X. R. Shi Q. Liu B. Q. Lei Y. D. Tang H. H. Zhang S. Z. (2021). Bacterial dynamics during the processing of nuodeng Dry-cured Ham. *Sci. Technol. Food Industry* 42 83–89. 10.13386/j.issn1002-0306.2020030129

[B44] Wang Y. B. Li F. Chen J. Sun Z. H. Wang F. F. Wang C. (2021). High-throughput sequencing-based characterization of the predominant microbial community associated with characteristic flavor formation in Jinhua Ham. *Food Microbiol.* 94:103643. 10.1016/J.FM.2020.103643 33279069

[B45] Wang Y. R. Liao H. Zhao H. J. Zhang Z. D. Guo Z. (2018). Evaluation of the bacterial diversity in cured fish samples collected from Enshi by PCR-DGGE and MiSeq high-throughput sequencing. *Modern Food Sci. Technol.* 34 208–213. 10.13982/j.mfst.1673-9078.2018.11.031

[B46] Wen K. Y. Wang Y. Wen P. C. Zhu Y. Yang M. Zhang Z. M. (2020). Study on microbial community structure in Sichuan traditional bacon. *Food Fermentat. Industr.* 46 36–42. 10.13995/j.cnki.11-1802/ts.022133

[B47] Wu W. H. Zhou Y. Wang G. Y. Zhu R. J. Ge C. R. Liao G. Z. (2020). Changes in the physicochemical properties and volatile flavor compounds of dry-cured Chinese Laowo ham during processing. *J. Food Process. Preserv.* 44:e14593. 10.1111/jfpp.14593

[B48] Wu Y. Y. Qian X. X. Li L. H. Chen S. J. Deng J. C. Li C. S. (2017). Microbial community diversity in dried-salted fish during processing revealed by Illumina MiSeq sequencing. *Food Sci.* 38 1–8. 10.7506/spkx1002-6630-201712001

[B49] Xie K. Yu X. F. Zheng H. S. Zong K. Lian Y. Q. Liu G. Q. (2013). Analysis of dominant microbial species in cantonese sausage by independent culture and PCR-DGGE technology. *Food Sci.* 34 157–160.

[B50] Xu Y. S. Zang J. H. Regenstein J. M. Xia W. S. (2020). Technological roles of microorganisms in fish fermentation: a review. *Critic. Rev. Food Sci. Nutr.* 61 1000–1012. 10.1080/10408398.2020.1750342 32292041

[B51] Yi L. B. Su G. R. Hu G. Peng Q. Z. (2017). Diversity study of microbial community in bacon using metagenomic analysis. *J. Food Saf.* 37:e12334. 10.1111/jfs.12334

[B52] You G. Wu Y. Y. Li L. H. Yang X. Q. Qi B. Chen S. J. (2015). Effect of inoculating compound lactic acid bacteria on microbial, nitrites and nitrosamines of salted fish. *South China Fish. Sci.* 11 109–115. 10.3969/j.issn.2095-0780.2015.04.016

[B53] Yu C. Q. Yao D. Man Y. G. Wang C. Y. (2010). Harm and control on biogenic amines of fermented meat products. *Meat Res.* 31 41–45. 10.3969/j.issn.1001-8123.2010.01.013

[B54] Zeng L. B. Xiong S. B. Wang L. (2009). Changes in microbe quantity and physico-chemical properties during processing of cured silver carp. *Food Sci.* 30 54–57. 10.3321/j.issn:1002-6630.2009.03.011 30704229

[B55] Zhang C. X. He Z. F. Li H. J. (2017). Research of bacteriocins from lactic acid bacteria and their applications in preservation of meat products. *Food Fermentat. Industr.* 43 271–277. 10.13995/j.cnki.11-1802/ts.013971

[B56] Zhang Q. Ding Y. C. Gu S. Q. Zhu S. C. Zhou X. X. Ding Y. T. (2020). Identification of changes in volatile compounds in dry-cured fish during storage using HS-GC-IMS. *Food Res. Intern.* 137:109339. 10.1016/J.FOODRES.2020.109339 33233050

[B57] Zhang S. Y. Tang N. Huang P. Zhou Y. Xu B. C. Li P. J. (2019). Isolation, identification and tolerance characteristics of microorganisms from Weining Ham. *Meat Res.* 33 12–17. 10.7506/rlyj1001-8123-20190625-147

[B58] Zhao D. B. Wang L. Y. Fu Y. Wang J. H. Tan C. X. Wang M. M. (2017). Study on strain screening, identification and physical and chemical properties characteristics of bacteriocin- producing lactic acid bacteria in Nanjing dry-cured duck. *Meat Industry* 37, 19–23. 10.3969/j.issn.1008-5467.2017.01.007

[B59] Zhou H. M. Zhang S. L. Zhao B. Li S. Pan X. Q. Ren S. (2018). Effect of starter culture mixture of *Staphylococcus xylosus* and *S. carnosus* on the quality of dry-cured meat. *Food Sci.* 39 32–38. 10.7506/spkx1002-6630-201822006

[B60] Zou Y. L. Liu S. Y. Wang G. Y. Pu Y. H. Ge C. G. Liao G. Z. (2020). Analysis of fungal community structure in Xuanwei ham by PCR-DGGE. *Food Fermentat. Industr.* 46 269–274. 10.13995/j.cnki.11-1802/ts.022394

